# Effects of maturity on physicochemical properties of Gac fruit (*Momordica cochinchinensis* Spreng.)

**DOI:** 10.1002/fsn3.291

**Published:** 2015-10-06

**Authors:** Xuan T. Tran, Sophie E. Parks, Paul D. Roach, John B. Golding, Minh H. Nguyen

**Affiliations:** ^1^ School of Environmental and Life SciencesUniversity of NewcastleOurimbahNSW2258Australia; ^2^ NSW Department Primary IndustriesCentral Coast Primary Industries CentreOurimbahNSW2258Australia; ^3^ School of Science and HealthUniversity of Western SydneyPenrithNSW2751Australia

**Keywords:** *β*‐carotene, Gac fruit, lycopene, maturation, oil content

## Abstract

The aril around the seeds of Gac fruit is rich in fatty acids and carotenoids (lycopene and *β*‐carotene). Understanding how these qualities are affected by fruit maturity at harvest may identify indices for quality assessment. Some physical and chemical properties of Gac fruit were determined for fruit harvested between 8 and 16 weeks after pollination (WAP). Fruit respiration rates and ethylene production rates were assessed after harvest and up to 20 days in storage at 20°C. Fruit harvested at 14 WAP had the highest oil (0.27 ± 0.02 g/g DW), lycopene content (0.45 ± 0.09 mg/g FW), and *β*‐carotene content (0.33 ± 0.05 mg/g FW) which declined by 16 WAP. External skin color and aril TSS were indicative of oil and carotenoid contents in aril. Skin color, TSS and potentially firmness were good indices of fruit quality. Harvesting less mature fruit at 12 WAP would be practical as the fruit were firmer and more capable of transport; however, quality during postharvest ripening may be limited. Fruits continued to ripen after they were harvested and an ethylene peak in the least mature fruit may reflect a climacteric behavior but this needs further investigation.

## Introduction

Gac (*Momordica cochinchinensis* Spreng.) is a tropical fruit that is used as a food and as traditional medicine in East and Southeast Asia (Iwamoto et al. 1985). The red aril which surrounds the seeds of mature Gac fruit is consumed in the traditional recipe Vietnamese “Xoi Gac”, a popular sticky rice dish that is eaten at weddings and New Year celebrations. In addition, the immature green fruit is also used as a vegetable in Thailand (Kubola and Siriamornpun 2011) and India (Joseph and Bharathi 2008). The ripe aril has a very high content of lycopene and *β*‐carotene and is a natural red colorant. The highest content of lycopene in fresh aril in fully ripe fruit was reported as 3.728 mg/g while *β*‐carotene levels were 0.379 mg/g (Nhung et al. 2010). These levels are at least five times higher than the lycopene content that is reported in the tomatoes (Rao and Agarwal 1999; Lenucci et al. 2009) and eight times higher than the *β*‐carotene content reported in the carrots (Cefola et al. 2012).

External subjective measures of fruit quality such as skin color, firmness, and fruit size influence consumer acceptance of some fruits, but many consumers are also interested in nutritional quality (Iglesias et al. 2012). For Gac fruit, the major consumer quality attributes include fruit size and weight, skin color, firmness, aril thickness, and color. Traditionally, Vietnam consumers generally prefer Gac fruits to be about 1.2–2 kg with good firmness and to have red skin with thick dark‐red aril around the seeds. Fruits that are very soft are considered “too old” and of a poorer quality, which attract lower prices. Understanding the relationships between the external measures of fruit quality such as skin color and firmness and the nutritional qualities of the aril inside the fruit will lead to simple and quantifiable indicators of fruit quality.

The quality of Gac fruit is strongly affected by its maturity at harvest. Fruit harvested at the full maturity level has shown to contain the highest concentration of carotenoids (lycopene and *β*‐carotene content) in the aril compared with green and semi‐ripe fruits (Nhung et al. 2010; Kubola and Siriamornpun 2011). A previous study has also shown that fruit size can affect the thickness of aril which increases with increasing fruit size (Parks et al. 2013). However, these previous studies are limited as they only considered up to three stages of fruit maturity, which were poorly defined and did not explore the relationships between other important physical characteristics such as fruit weight or skin color and the nutritional quality of the aril. Using well‐defined maturity stages, such as the number of weeks after the time of pollination (WAP), provide a clear definition of maturity and allow a clear comparison between multiple maturity stages.

In this study, the relationships between a number of physical properties (fruit weight, size, skin color, and flesh firmness) and chemical properties (concentration of carotenoids, total soluble solid (TSS), pH, titratable acidity (TA), and oil content) of Gac fruit harvested at five maturity stages were made. The aim of this study was to determine if any of these properties could be used as an indirect measure of aril quality. Indirect measures of quality would be of great benefit to growers, consumers, and processors to make informed decisions about when to harvest, sell, and consume Gac fruits with an optimum nutritional content.

## Materials and Method

### Crop production

A Gac crop was produced in greenhouses at the NSW Department of Primary Industries at Ourimbah, NSW, Australia (151°22′E, 33°21′S). The 18 plants (12 female and six male plants) were grown from seed in February 2013. The seed was sourced from fruit grown locally in Sydney, Australia. The environmental parameters and growing conditions for the Gac crop are described by Parks et al. (2013). The greenhouse temperature and the relative humidity were maintained between 18 to 25°C and 60 to 80% respectively. A hydroponic system provided water and fertilisers with a complete nutrient solution with a target electrical conductivity (EC) of 1.2 dS/m, and a pH of 5–6.5. At the flowering stage, the target EC of the nutrient solution was increased to 2.4 dS/m. The female flowers were hand pollinated using fresh pollen collected from the male flowers.

### Fruit harvest

Gac fruits were harvested, between 12 November 2013 (first fruit) and 16 March 2014 (last fruit) from 12 female plants at five stages of maturity: 8, 10, 12, 14, and 16 weeks after the day of pollination (WAP) and each stage included eight fruits. The fruit characteristics at the five stages of maturity are described in Table 1.

**Table 1:** Description of Gac fruit at five stages of maturity

Stages	WAP	Description of maturity stages
M1	8	Fully green skin, white pulp, light yellow aril
M2	10	Green skin, spines turning yellow at the top of fruit, white pulp, yellow or pink aril
M3	12	Semi‐ripe, skin starting to yellow or orange in patches, light yellow pulp, red aril
M4	14	Ripened, fully orange or red skin, yellow pulp, red aril
M5	16	Fully ripe, dark‐red skin, dark yellow pulp, dark‐red aril

WAP, weeks after pollination.

Two to four fruits were harvested per plant and every fruit from the same plant was assigned to a different stage of maturity. The eight fruit for each maturity stage were sampled on different dates with three fruits used for quality measurements (weight, size, color, firmness, TSS, pH, oil content, lycopene, and *β*‐carotene content) and five fruits used for respiration and ethylene production measurements at 20°C for up to 20 days after harvest. The Gac fruits were all harvested in the morning and delivered to the laboratory within 30 min for analysis.

### Fruit morphology and moisture content

The weight of the whole fruit and its components (skin, pulp, aril and seed (mature black seed and immature white seed)) were determined using an electronic balance. Fruit length (polar axis‐distance between the apex and the stem) and fruit diameter (the maximum width perpendicular to the polar axis) were measured. Skin color was measured using a Minolta Chroma Meter CR‐400/410 (Minolta Corp, Osaka, Japan) where 10 measurements (L*, a*, b*) were taken along the equatorial axis of each fruit for three fruits per maturity stage. The color parameters chroma (C* = (a*^2^+b*^2^)^1/2^) and hue angle (h° = arctan (b*/a*)) were calculated for fruit skin color as described by McLellan et al. (1995). The flesh firmness was determined using a drill‐mounted penetrometer (Facchini, Alfonsine, Italy) and measuring the force to manually lower a flat penetrometer tip (8‐mm diameter) under constant force to penetrate into the fruit to a depth of 8 mm, corresponding to a mark inscribed on the shaft of the probe (Harker et al. 1996). The value of fruit firmness was an average of 10 points per fruit for three replicate fruits. The results were expressed as load in kilograms force (kgf). The moisture content of the fruit components was measured by drying at 70°C in a vacuum oven (Vord‐46OD; Australasian Scientific Marketing Group, Kotara, NSW, Australia) until a constant weight was obtained.

### Respiration rate and ethylene measurements

Approximately 1 h after harvest, the respiration rate and the ethylene production of the fruit were determined by measuring the concentration of CO_2_ and ethylene accumulated from each Gac fruit placed in a 4 L sealed container previously equilibrated for 24 h in a controlled room at 20°C and 80–85% RH for at least 30 min. Five replicate fruits were used for this experiment. The respiration rate and the ethylene production rates were determined daily for each fruit for 20 days after harvest at 20°C.

#### Respiration rate

A gas sample (5 mL) of the headspace was withdrawn from each sealed container, containing a single Gac fruit, the level of CO_2_ within the sample were measured with ICA gas analyser (ICA 40 system; Tonbridge, Kent, UK) and the respiration rate were calculated.

#### Ethylene production

A gas sample (1 mL) of the headspace was withdrawn from each sealed containers, containing a single Gac fruit, and the concentration of ethylene determined with Gas Chromatograph (Gow‐Mac‐580 Instrument Co., Bethlehem, PA, USA) equipped with a stainless steel column (190 mm × 216 mm) packed with activated alumina (80–100 mesh; Alltech, Sydney, NSW, Australia) and a flame ionization detector. The operation temperatures were 70, 90, and 100°C for the injector, column and detector respectively. The gas flow rates were 20, 30, and 300 mL/min for nitrogen, hydrogen and air respectively.

Ethylene production rate for each fruit were determined daily for 20 days after harvest at 20°C.

### The aril quality of the Gac fruit

#### Total soluble solids, pH, and titratable acidity of the aril

The red aril was separated from the seed then blended and filtered with cheese cloth to determine TSS, pH, and TA on the juice. The TSS was measured using a digital refractometer (Atago Co.Ltd, Tokyo, Japan) and the pH was determined at room temperature using a pH meter (Hanna Instruments Inc., Woonsocket, RI, USA). The TA was measured using an automatic titrator (Mettler Toledo T50, Schwerzenbach, Switzerland) against 0.1 N NaOH to an end‐point of pH 8.2 and the results were expressed as g citric acid (CA) per 100 mL.

### HPLC analysis of β‐carotene and lycopene content

The content of *β*‐carotene and lycopene in Gac aril was evaluated at each maturity stage using high performance liquid chromatography (HPLC) as described by Kha et al. (2013b). Two grams of fresh aril were crushed and extracted with 60 mL of ethanol: hexane (4:3 v/v) at room temperature until there was no color left in the aril material. The extracted solution was filtered using a 0.45 *μ*m syringe filter (Grace Davison, Rowville, Vic., Australia) prior to injection onto the Agilent 1200 HPLC system and Shimadzu LC‐10AD HPLC system equipped with a Luna C18 (100 × 46 mm) coupled to a Jupiter C18 (250 × 46 mm) column (Phenomenex, Lane Cove, NSW, Australia). The mobile phase consisted of acetonitrile (ACN), dichloromethane (DCM) and methanol (MeOH) (5:4:1, v/v/v). The injection volume was 20 *μ*L, the flow rate was 1.0 mL/min, and detection was at 450 nm. The *β*‐carotene and lycopene were quantified based on the retention times and standard curves of authentic standards (Sigma‐Aldrich, Castle Hill, NSW, Australia) and their content was expressed as mg/g of fresh aril weight (FW).

### Determination of total oil content

The total oil content of the Gac fruit aril was measured using the Soxhlet extraction method as described by Kha et al. (2013b). First, 200 g of the fresh aril was dried using microwave oven (Model R42BST; Sharp Corporation, Artarmon, Australia) and 3 g of the dried‐aril was placed in a cellulose thimble, which was inserted into the Soxhlet apparatus and extracted with 300 mL of boiling hexane until the sample was colorless. The extracted Gac sample was left at room temperature for 15 min in a fumehood to evaporate any remaining hexane and then dried at 70°C in a vacuum oven (Vord‐46OD; Australasian Scientific Marketing Group) until a constant weight was obtained. The oil content was then calculated by difference and expressed as weight percentage (g/g of dry weight, DW).

### Statistical analyses

All chemical characteristic measurements were done in triplicate. The values were expressed as means ± standard deviations (SD) and statistical analyses were performed using the Statistical Package for the Social Sciences (SPSS) software version 22 (IBM Corp., Armonk, NY, USA). Analysis of variance (ANOVA), followed by the least significant difference (LSD), post hoc test, was performed to determine statistical significance between the five stages of maturity (M1 to M5). Pearson's correlation test was used to determine whether there were any significant associations between the mean values for the variables. Statistical significance was given at *P* < 0.05.

## Results

### Fruit morphology

As expected, as the Gac fruits grew on the plant, the fruit from each maturity stage were larger and heavier. The results presented in Table 2 show that the fruit harvested at stages M4 and M5 were larger (length and diameter) and heavier compared to fruit harvested at stage M1. Gac fruit weight increased during the developmental stages, increasing 66% from stage M1 to stage M5. Although the fruit length and diameter increased as the fruit matured, the ratio of length/diameter remained similar during the five stages at 0.5 (Table 2).

**Table 2:** Physical properties of Gac fruit at five maturity stages

FruitAttributes	Fruit maturity stages				
	M1	M2	M3	M4	M5
Weight (g)	1040.42 ± 294^a^	1276.62 ± 269^ab^	1586.64 ± 174^ab^	1665.76 ± 185^b^	1729.74 ± 227^b^
Length (cm)	21.33 ± 1.22^a^	22.67 ± 1.44^ab^	24.50 ± 1.67^ab^	25.33 ± 1.44^b^	26.33 ± 1.56^b^
Diameter (cm)	42.50 ± 3.00^a^	44.67 ± 2.78^a^	48.67 ± 1.44^b^	49.17 ± 1.78^b^	50.83 ± 2.22^b^
Length/diameter	0.50 ± 0.01^a^	0.51 ± 0.00^a^	0.50 ± 0.02^a^	0.51 ± 0.02^a^	0.52 ± 0.01^a^
Total seeds	32.67 ± 9.11^a^	33.00 ± 2.67^a^	36.00 ± 4.67^a^	37.67 ± 1.56^a^	34.00 ± 8.67^a^
Immature seeds	32.00 ± 9.33^b^	4.67 ± 6.22^a^	3.00 ± 1.33^a^	4.67 ± 2.44^a^	2.33 ± 1.56^a^
Mature seeds	0.67 ± 0.89^a^	28.33 ± 4.89^b^	33.00 ± 4.67^b^	33.00 ± 2.00^b^	31.67 ± 9.78^b^
Color					
a*	−18.25 ± 3.72^a^	−8.14 ± 6.83^ab^	−4.76 ± 6.12^b^	40.11 ± 3.60^c^	44.08 ± 6.07^c^
b*	39.87 ± 5.14^a^	40.27 ± 4.45^a^	49.03 ± 5.67^ab^	51.55 ± 11.17^ab^	58.66 ± 6.06^b^
L*	37.39 ± 1.28^a^	38.50 ± 3.11^a^	46.06 ± 3.22^a^	42.17 ± 3.83^a^	43.05 ± 5.55^a^
*h°*	114.38 ± 2.87^c^	100.97 ± 9.43^bc^	96.41 ± 7.20^b^	51.52 ± 3.20^a^	53.02 ± 5.54^a^
Firmness (kgf)	>15	>15	9.67 ± 3.30^b^	2.96 ± 0.10^a^	2.28 ± 0.09^a^

The values are means ± SD (*n* = 3) and those not sharing the same letter superscripts in a row are significantly different (*P* < 0.05) as determined using the ANOVA and the LSD post hoc test. The different stages of fruit maturity (M1 to M5) are defined in Table 1.

The development of the seeds within the Gac fruit was affected by the harvest maturity stage, where there was a significant difference in the number of immature white seeds relative to fully mature black seeds between stage M1 and the other stages (Table 2). At stage M1, the seeds were almost all white (98%) and they were not completely developed, whereas at the other stages they were mostly black (86–93%) and well formed.

The external color of the Gac fruit skin as measured with the Minolta color meter showed a distinct progression through the five distinct stages described in Table 1. Skin color at M1 was dark green but changed as the fruit matured to green with some yellowing (M2), to yellow and orange (M3), to orange and red (M4), and finally dark red (M5). The changes of skin color were clearly reflected by the Chroma meter a* value (which indicates “greenness” when it is negative and “redness” when it is positive), increasing progressively from being negative at stage M1, M2 and M3 to being highly positive at stage M4 with no further increase at the last stage (M5) (Table 2). Where there were significant increases between stage M1, M3 and stage M4, fruit skin changed from fully green at M1 to semi‐ripe at M3 and fully ripen at M4. The L* value (which indicates the lightness or darkness of fruit), remained stable at the five maturity stages and the positive values indicated that the Gac fruit remained fairly light in color throughout. Conversely, the hue angle (h° value, which integrates both a* and b* values) decreased significantly between stage M3 (in the yellow range) and M4 (in the red range). There was no hue angle color differences between M1 to M2 (in the green range) and between M4 to M5 (in the red range).

Flesh firmness could not be determined with the M1 and M2 stages as the fruits were too hard (>15 kgf), the maximum value on the penetrometer. However, during the growth and maturity, flesh firmness declined by ≥70% between the M3 stage and the M4 and M5 stages (Table 2), whereby the final maturity stage (M5), the fruit were very soft (2 kgf).

The relative weights of the four main fruit components (skin, yellow pulp, aril, and seeds), as a percentage of the whole fruit weight, and their moisture content at the five different maturity stages are presented in Table 3. The results show what the pulp made up the highest component of the fruit (36.2–43.9%) and whereas the seeds comprised were the lowest component (9.3–12.2%). The aril is the component of most interest, accounted for approximately 25–30% of the total fruit weight whist the skin contributed 14–19% to the total weight of the fruit. The results showed that although the total weight increased during growth (Table 2), the relative amounts of the fruit components were not affected by the maturity stages (Table 3).

**Table 3:** The components of Gac fruit and moisture contents at five maturity stages (*n* = 3)

Parameters (%)	Maturity stages				
	M1	M2	M3	M4	M5
Percentage of fruit					
Skin	19.08 ± 5.39^e^	16.86 ± 4.80^e^	15.70 ± 2.49^e^	13.89 ± 2.32^e^	15.16 ± 0.17^e^
Pulp	38.00 ± 2.11^f^	36.99 ± 3.35^f^	36.20 ± 1.23^f^	43.86 ± 4.99^f^	42.74 ± 3.85^f^
Aril	24.92 ± 1.57^g^	26.64 ± 2.35^g^	29.17 ± 2.21^g^	30.00 ± 3.78^g^	29.50 ± 4.37^g^
Seed	12.24 ± 1.85^h^	9.77 ± 1.65^h^	10.20 ± 1.52^h^	9.33 ± 0.67^h^	9.84 ± 1.73^h^
Moisture content					
Skin	86.94 ± 0.33^c^	85.34 ± 0.89^c^	78.11 ± 1.81^a^	83.97 ± 0.29^c^	79.11 ± 1.89^b^
Pulp	93.96 ± 0.06^d^	91.15 ± 0.23^c^	89.61 ± 0.73^b^	91.42 ± 0.17^c^	88.68 ± 0.28^a^
Aril	89.66 ± 0.37^e^	76.28 ± 0.46^b^	78.91 ± 0.75^c^	82.31 ± 0.25^d^	72.51 ± 1.61^a^
Seed	64.77 ± 1.17^d^	30.65 ± 0.70^b^	37.90 ± 2.11^c^	30.36 ± 0.56^b^	25.91 ± 0.98^a^

The values are means ± SD (*n* = 3) and those not sharing the same letter superscripts for the proportion of fruit or for the moisture content are significantly different (*P* < 0.05) as determined using the ANOVA and the LSD post hoc test. The different stages of fruit maturity (M1 to M5) are defined in Table 1.

In contrast, the moisture content of the five components was influenced by the harvest maturity stage. A significant difference in the moisture content of the aril was observed at all five maturity stages, where the moisture content of the four components were higher at stage M1 and decreased through the other stages to be 9% lower for the skin, 5.6% lower for the pulp, 19% lower for the aril, and 60% lower for the seeds at stage M5.

### Physiological properties

#### Respiration rate

**Figure 1:**
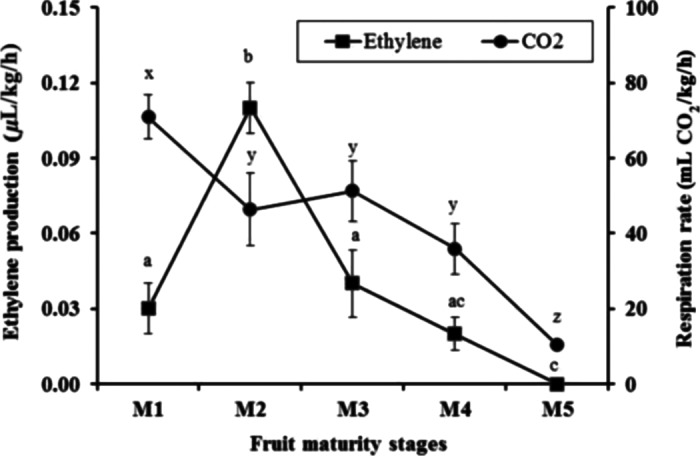
The respiration rate and ethylene production of Gac fruits harvested at five stage of maturity. Measurements were taken 1 day following harvest. The values are means ± SD (*n* = 5) and those not sharing a letter (a–d for ethylene, x–z for respiration) are significantly different (*P* < 0.05).

The results of the respiration rates of the Gac fruit from the different harvest maturity stages are presented in Figure 1 and show that the respiration rate of fruit at the first day after harvest depended on the stage of maturity. The results showed that fruit from the early harvest maturities had higher respiration rates than those fruit at the more mature stages (Fig. 1). The maximum respiration rate was obtained on the first day after harvest for the M1 stage (71 mL CO_2_/kg/h) and it was lower for the M2 (46 mL CO_2_/kg/h), M3 (51 mL CO_2_/kg/h), M4 (36 mL CO_2_/kg/h), and M5 stages (10 mL CO_2_/kg/h).

**Figure 2:**
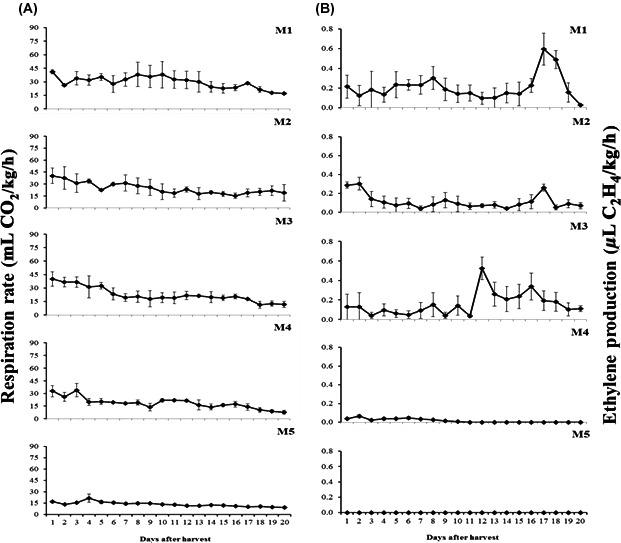
The respiration rate and ethylene production of Gac fruit during 20 days after harvest at the five stages of maturity: (A) respiration rate; (B) ethylene production. The values are means ± SD; (*n* = 5).

The respiration rates over time after fruit harvest from the different harvest maturities are presented in Figure 2 and show that the production of carbon dioxide generally declined during postharvest storage at 20°C.

#### Ethylene production

The levels of ethylene production on the first day after harvest in fruit at the M2 stage were higher than all other maturity stages (Fig. 1). Conversely, the ethylene production rates of fruit harvested at the final maturity stage, M5 was the lowest ethylene production rate (Fig. 1).

During the 20 days of storage at 20°C, a peak of ethylene production was observed on different days for different maturity stages (Fig. 2). At stage M1, the highest level of ethylene (0.59 *μ*L C_2_H_4_/kg/h) was obtained 17 days after harvesting. Similarly, the peak ethylene production rate for the M2 stage (0.26 *μ*L C_2_H_4_/kg/h) was observed 17 days after harvest (Fig. 2). Unfortunately, skin color was not monitored during the postharvest storage life at 20°C, but it was observed that the color of fruit's skin had completely changed from green to yellow at day 17 at the M1 stage.

In contrast, the highest concentration of ethylene for the M3 stage was 0.52 *μ*L C_2_H_4_/kg/h 12 days after harvest (Fig. 2). Very low levels were observed for the M4 stage, with a peak of 0.06 *μ*L C_2_H_4_/kg/h 2 days after harvesting and no ethylene production was detected for the M5 stage (Fig. 2).

### Fruit quality

#### Total soluble solids and pH of the aril

A close relationship between harvest maturity and TSS in the aril of the Gac fruit was observed. The results in Table 4 show that the TSS of the Gac aril was significantly different at each of the five maturity stages with the highest levels of TSS measured at the M4 stage. There was an increase in the TSS of the Gac aril from the M1 stage (4.9%) to the M4 stage (15.8%) but these levels declined to 13.9% at the final maturity stage (M5 stage) (Table 4).

**Table 4:** Chemical properties of Gac aril juice at different maturity stages

Attributes	Fruit maturity stages				
	M1	M2	M3	M4	M5
Total soluble solid, TSS (°Brix)	4.93 ± 0.38^a^	6.30 ± 0.07^b^	12.43 ± 0.42^c^	15.80 ± 0.13^d^	13.90 ± 0.87^e^
pH	6.54 ± 0.11^a^	6.74 ± 0.10^a^	6.56 ± 0.18^a^	6.73 ± 0.13^a^	6.52 ± 0.14^a^
TA (g CA per 100 mL)	0.24 ± 0.01^bc^	0.18 ± 0.01^a^	0.29 ± 0.05^c^	0.26 ± 0.01^bc^	0.21 ± 0.01^ab^
TSS:TA	20.88 ± 1.68^a^	36.11 ± 1.97^b^	44.64 ± 6.71^b^	59.72 ± 0.97^c^	65.97 ± 2.87^c^

The values are means ± SD (*n* = 3) and those not sharing the same letter superscripts in a row are significantly different (*P* < 0.05) as determined using the ANOVA and the LSD post hoc test.

As the TSS of the Gac aril increased during maturation, the pH of the aril remained stable (Table 4). Titratable acidity (TA) were generally low across all the maturity stages, but were lowest at M2 stages then followed by an increase at semi‐mature stage (M3). A gradual decrease in TA was observed with advancing fruit maturity (M3 to M5). The TSS/TA ratio increased from 20.9 at the M1 stage to 66.0 at the M5 stage.

#### Oil, lycopene and β‐carotene contents in the aril

The harvest maturity stages strongly influenced the oil content of Gac aril (Table 5). The oil content increased significantly from 0.03 g/g DW at M1 stage to a maximum level of 0.27 g/g DW at M4; the M5, the oil content was 0.25 g/g DW and was not different from the values measured at M3 and M4 (Table 5).

**Table 5:** The oil, lycopene, and *β*‐carotene content of Gac fruit at the five maturity stages

Attributes	Fruit maturity stages				
	M1	M2	M3	M4	M5
Oil content (g/g DW)	0.03 ± 0.01^a^	0.12 ± 0.01^b^	0.23 ± 0.02^c^	0.27 ± 0.02^c^	0.25 ± 0.01^c^
Lycopene (mg/g FW)	0.02 ± 0.00^a^	0.23 ± 0.04^b^	0.31 ± 0.04^b^	0.45 ± 0.09^c^	0.28 ± 0.00^b^
*β*‐carotene (mg/g FW)	0.02 ± 0.00^a^	0.13 ± 0.04^b^	0.22 ± 0.06^cd^	0.33 ± 0.05^d^	0.21 ± 0.02^bc^
Total carotenoids (mg/g FW)	0.05 ± 0.01^a^	0.36 ± 0.06^b^	0.53 ± 0.06^b^	0.78 ± 0.12^c^	0.49 ± 0.02^b^

The values are means ± SD (*n* = 3) and those not sharing the same letter superscripts in a row are significantly different (*P* < 0.05) as determined using the ANOVA and the LSD post hoc test; FW, fresh aril weight.

The content of lycopene and *β*‐carotene in the aril increased with fruit maturity, similar to the increase for oil content (Table 5). At the fully green stage, the lycopene and *β*‐carotene contents were at their lowest values (0.02 and 0.02 mg/g FW respectively). The lycopene content was highest at the M4 stage (0.45 mg/g FW) before it decreased to at the M5 harvest maturity stage (0.28 mg/g FW). Similarly, the *β*‐carotene content increased at the M4 stage (0.33 mg/g FW) but decreased at the M5 stage (0.21 mg/g FW). While, the lycopene content in the aril was not different at the three stages M2, M3, and M5 and the *β*‐carotene content at the M5 stage was not different from its contents at the M3 and M4 stages. The total carotenoids content (total of lycopene and *β*‐carotene content) was highest at M4 stage (0.78 mg/g FW), where there were no differences between the M2, M3, and M5 maturity stages.

### Multivariate analysis

The relationships between the morphological properties and the quality parameters of the Gac fruit during fruit development were investigated using the Pearson correlation and the results are presented in Table 6. The results showed that there were positive correlations (*r* > 0.65, *P* < 0.01) between the fruit size parameters (weight, length, and diameter) and the aril TSS and oil content. Fruit diameter possessed the highest correlations (*r* > 0.70, *P* < 0.01). Fruit length and diameter also had strong inverse correlations with the respiration rate after harvest (*r* = −0.68 and *r* = −0.67, *P* < 0.01, respectively) but there was no correlation with ethylene production after harvest.

**Table 6:** Correlations between the morphological properties and the quality parameter of the Gac during fruit development

Parameters	1	2	3	4	5	6	7	8	9	10	11	12	13	14
1. Weight	1													
2. Length	0.952**	1												
3. Diameter	0.983**	0.948**	1											
4. L*	0.553*	0.618*	0.611*	1										
5. a*	0.569*	0.637*	0.614*	0.334	1									
6. Hue angle	−0.535*	−0.580*	−0.573*	−0.303	−0.985**	1								
7. Firmness	−0.043	−0.043	−0.152	0.261	−0.884**	0.893*	1							
8. CO_2_	−0.617*	−0.676**	−0.671**	−0.292	−0.939**	0.912**	0.832**	1						
9. C_2_H_4_	−0.333	−0.335	−0.410	−0.129	−0.569*	0.529*	0.892**	0.553*	1					
10. TSS	0.678**	0.671**	0.709**	0.413	0.817**	−0.841**	−0.539	−0.776**	−0.568*	1				
11. TA	0.057	−0.049	0.093	0.159	0.047	−0.083	0.278	0.105	−0.442	0.423	1			
12. Oil	0.686**	0.657**	0.710**	0.421	0.736**	−0.780**	−0.240	−0.719**	−0.363	0.941**	0.352	1		
13. Lycopene	0.618*	0.575*	0.599*	0.456	0.638*	−0.705**	−0.032	−0.565*	−0.103	0.810**	0.175	0.862**	1	
14. *β*‐carotene	0.553*	0.477*	0.578*	0.385	0.575*	−0.740**	−0.422	−0.655**	−0.318	0.835**	0.281	0.858**	0.855**	1

*P < 0.05 (2‐tailed); **P < 0.01 (2‐tailed).

There was a positive correlation between the color of the skin fruit (a* value) and the quality indices (TSS, oil content, lycopene, and *β*‐carotene, *r* > 0.575, *P* < 0.05) while the inverse correlation between a* value and firmness (*r* = −0.884, *P* < 0.01) and respiration rate (*r* = −0.939, *P* < 0.01) were observed. Significant (*P* < 0.01) negative correlation was observed between TSS and respiration rate (*r* = −0.776, *P* < 0.01).

Strong relationships (*P* < 0.01) between the TSS of the aril and the content of oil, lycopene and *β*‐carotene in the aril were observed (oil: *r* = 0.941, lycopene: *r* = 0.810, *β*‐carotene: *r* = 0.835). Although significant (*P* < 0.05), the correlations between the fruit size parameters (weight, length, and diameter) and the lycopene and *β*‐carotene content of the aril were less strong (*r* < 0.62) than for the aril TSS and oil content. There was an inverse correlation between the hue angle and the TSS, the oil, lycopene, and *β*‐carotene content of the aril (TSS: *r* = −0.84, oil: *r* = −0.78, lycopene: *r* = −0.71, *β*‐carotene: *r* = −0.74, *P* < 0.01) and this showed that the TSS, oil, lycopene, and *β*‐carotene content of the aril increased as the fruit matured.

## Discussion

This study clearly demonstrated some of the important changes in several physical and chemical properties in Gac fruit harvested at increasing stages of maturity and provides the first comprehensive account of maturation and ripening in Gac fruit. Ripening of Gac fruit was defined by the changes of skin color (green to orange and red), aril color (yellow to red), pulp color (white to yellow), seed color (white to black), and firmness of fruit (firm to soft). Ripening commenced on the vine in the fruit harvested at a mature stage (M3) and following harvest in less mature fruit (M2). The appearance of yellowing on the fruit skin spines (M2) was a sign that the maturation process had commenced. Fruit harvested at this stage of maturity had a prominent level of ethylene production relative to the other fruit maturities (Fig. 1). For fruits harvested green (M1), a peak of ethylene production occurred 17 days following harvest (Fig. 2). This production peak is evidence of ethylene climacteric behavior but this needs further investigation as respiration rates did not peak and were not characteristic of climacteric fruit. Nonetheless, respiration declined during storage at all maturity stages. Fruit rots did not occur at any stage of the experiment and thus did not exacerbate ethylene or respiration observations. Fruit ripening was characterized by fruit softening and increases in TSS, oil, and carotenoid contents.

The proportion of aril did not change as a function of maturity or fruit size, the larger the fruit size, the greater the internal aril volume. The aril, the most utilized part of the fruit, and the other components declined in moisture content with maturity (Table 3). Although the proportion of aril did not relate to fruit size, these data contrasted to another Gac study with more limited data by Parks et al. (2013). This study suggests that the customer would not be disadvantaged by purchasing smaller fruit at prices based on weight. The total number of seeds was not related to fruit quality (Table 2). Mature and immature seed number did not relate to fruit quality for fruit between M2 to M5 stages and thus do not provide a good index of quality. A significant increase in mature seeds from M1 to M2 and corresponding decrease in immature seeds reflects the maturation of these seeds between these stages of fruit maturity. Further work is required to investigate the potential effect of the mature seed number on the storage life of Gac.

Between M3 and M4 stages, the firmness of Gac fruit significantly declined (>70%) and this suggests that this characteristic may be a good indicator of quality as M4 fruit was of the best quality. As fruit firmness could not be measured in M1 and M2, data were limited and more research is required to evaluate this hypothesis.

Fruit maturity at harvest had a strong effect on the carotenoid contents in the aril, where increasing carotenoids were measured in the later maturity stages. The highest lycopene (0.454 mg/g FW) and *β*‐carotene (0.326 mg/g FW) contents were obtained in M4 fruit. The most advanced stage of ripeness (completely dark‐red skin, M5 stage) had lower levels of carotenoids than completely orange fruit (M4 stage) indicating that the fruit were over‐ripening. Kha et al. (2013a) reviewed the carotenoid contents in Gac fruit reported the contents were variable. The carotenoid contents obtained in this study are within the range reported by others (Aoki et al. 2002; Ishida et al. 2004; Vuong et al. 2006; Nhung et al. 2010) but clearly showed differences in the stage of fruit maturity at harvest. However, other variables such as variety, techniques used for analysis, and variable production conditions will also have an effect on the quality and carotenoid contents and should be further evaluated.

The skin color of Gac fruit showed a positive correlation with quality indices (TSS, carotenoid and oil contents) (Table 6). Growers and consumers could use this simple parameter to identify fully mature fruits with high quality. Although objective parameters of color (Minolta) and firmness (penetrometer) did not significantly separate the two most mature categories, the difference in color was apparent to the eye highlighting the large variability in the Minolta color measurements. Thus skin color, a parameter already used by consumers, when choosing fruits for purchase or when deciding if a fruit is ready to eat (Opara et al. 2009) is appropriate for Gac fruit.

As more mature Gac fruits were harvested, the TSS of the Gac aril considerably increased from 4.9% Brix at M1 to 15.8% Brix at M4. Such increases in the TSS level are common with fruit maturation and it has been observed in many fruit types (Salvador et al. 2007; Wanitchang et al. 2010; Fawole Olaniyi and Opara 2013). In addition, is likely that TSS also increases in Gac fruit as it ripens after harvest. We have previously observed TSS in the Gac aril increase during storage for Gac fruit harvested at a stage between M3 and M4 (Xuan et al., unpublished data). As TSS is strongly correlated with the oil and carotenoid contents (*r* > 0.81; *P* < 0.01) (Table 6), the simple measurement of TSS with a refractometer maybe a practical and simple tool to use as an indicator of fruit quality. However, more research is required to verify this relationship in different varieties and growing situations.

The increase in the ratio TSS/TA of Gac aril juice during fruit maturation was similar to other fruits (Pinillos et al. 2011; Fawole Olaniyi and Opara 2013) and is also a reliable indicator of quality (Table 4). The TSS represents the total soluble solids such as sugars, acids and other components (Bailen et al. 2006). The relatively high level of TSS in Gac aril (15.8% Brix at M4) is interesting, as the informal assessment of taste of Gac fruit is not sweet (unpublished data) like other fruits with similar levels. Although taste is a complex interaction of many characteristics, this may reflect that sugar levels may be a small proportion of the TSS but this needs further investigation.

## Conclusions

This study has identified several measures that provide an index of maturity which relates to Gac fruit quality in terms of high contents of oil and carotenoids. The objective indices useful for identifying high‐quality fruit are skin color, TSS, the TSS: TA ratio and most likely firmness. Those measures identified as not being suitable are total seed number and TA. Gac fruits harvested at the M4 stage were ripe and soft, had completely orange or red skin with a yellow pulp and red aril. These were of the highest quality which could be considered best for consumption. However, we propose that Gac fruits can be picked at the M3 stage for transportation and distribution to the consumer. The fruit described at the M3 stage were semi‐ripe and firm with skin starting to yellow or orange in patches and with light yellow pulp and red aril. Whether or not fruits harvested at the M2 and M3 stage can be ripened in postharvest to obtain acceptable levels of carotenoids and oil contents needs to be clarified. In addition, confirming the climacteric nature of Gac fruit needs further investigation.

## Conflict of Interest

None declared.
